# Standard setting for a novel esophageal conduit questionnaire: CONDUIT Report Card

**DOI:** 10.1186/s41687-018-0073-2

**Published:** 2018-10-24

**Authors:** Minji K. Lee, Kathleen J. Yost, Karlyn E. Pierson, Amy J. Schrandt, Bobbie J. Skaare, Shanda H. Blackmon

**Affiliations:** 1 Mayo Clinic Robert D. and Patricia E. Kern Center for the Science of Health Care Delivery, Mayo Clinic, Rochester, MN USA; 2 Department of Health Sciences Research, Mayo Clinic, Rochester, MN USA; 3 Department of Surgery, Division of Thoracic Surgery, Mayo Clinic, Rochester, MN USA

**Keywords:** Esophageal cancer, Patient-reported outcomes, Cut scores, Standard setting, Modified Angoff methods

## Abstract

**Background:**

The purpose of this study was to establish the clinical thresholds for five domains (dysphagia, reflux, dumping-hypoglycemia, dumping-GI symptoms, pain) to support the use of the CONDUIT questionnaire as a screening tool to identify patients who might benefit from an educational or clinical intervention.

**Methods:**

A panel of 16 experts met to develop descriptions of “poor,” “moderate,” and “good” conduit performance. They were trained to use the modified and extended Angoff standard-setting method. Each judge provided item ratings that reflected borderline good and borderline moderate patients. The average item ratings were summed and transformed to a 0–100 scale to derive final cut scores. Panelist evaluation of the process and confidence with the rating tasks were collected.

**Results:**

Panelists expressed that the training on the method gave them information they needed to complete their assignment. Among other factors, their experience with patients was most influential on their ratings. On the 0–100 score scale, good/moderate cuts ranged from 7.2 to 20.8, and moderate/poor cuts ranged from 37.9 to 64.3, depending on domains and weights. Standard errors of one or both cut scores increased for dysphagia and dumping-GI with weighting.

**Conclusions:**

We described the selection and training of panelists and panelists’ evaluations of the processes they were asked to follow in detail to defend the cut scores. Further prospective validation studies are underway to compare cut scores from this study and clinicians’ judgments and further refine the categorization.

**Electronic supplementary material:**

The online version of this article (10.1186/s41687-018-0073-2) contains supplementary material, which is available to authorized users.

## Background

Quality of life (QOL) has been shown to deteriorate following esophageal reconstruction, with patients suffering multiple symptoms within six months [1–3]. Consistent with findings of others [4], our prior work noted that patients can benefit from periodic assessments to detect increased morbidity on the basis of subjective self-reports [5]. In this work, we have established five multi-item domains for score reporting after esophageal reconstruction on the novel questionnaire, Mayo **C**linic Esophageal Conduit **O**utcomes **N**oting **D**ysphagia/Dumping, and **U**nknown Outcomes with **I**ntermittent Symptoms Over **T**ime After Esophageal Reconstruction (CONDUIT) Report Card. The content of the CONDUIT Report Card was informed by extensive engagement with patients. See Lee et al. [5] for more details on its development. The five domains constitute dysphagia, reflux, dumping-gastrointestinal (dumping-GI) symptoms, dumping-hypoglycemia, and pain (Additional file 1). We also report scores on dyspnea from a previously established measure, Medical Research Council (MRC) breathlessness scale [6].

**Fig. 1:**
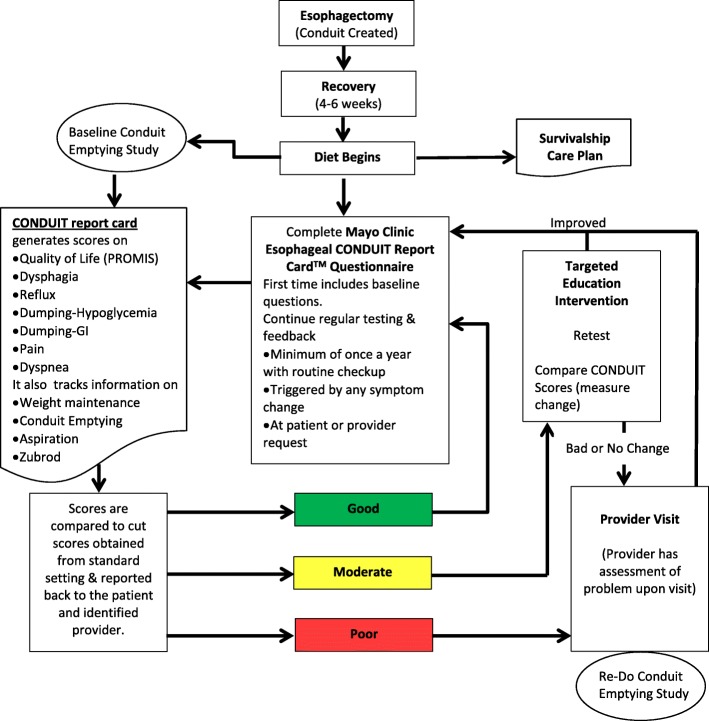
Flow diagram of how three performance categories from CONDUIT Report Card inform clinical activities

Scores for each new construct on the CONDUIT Report Card (CONDUIT henceforth) are on a 0 to 100 continuous scale (a higher score means higher level of the construct), which is useful for providing confidence bands around scores and for measuring changes in quality of life. However, to enhance clinical utility and inform care delivery, clinicians often prefer absolute values of scores classified into clinically distinct categories. For example, Eckardt Score [7] has four grades—0, 1, 2, and 3—to distinguish between levels of four major symptoms of achalasia. Vantrappen and Helleman’s classification [8] uses four classes—excellent, good, fair, and poor—to describe the conduit performance as a whole after treating achalasia. Dysphagia Grade [9] has five levels to describe severity of dysphagia. Other studies on related diseases used classifications such as “none,” “mild,” “moderate,” and “severe” [10] or “none,” “monthly,” “weekly,” and “daily” [11] to describe symptoms preoperatively and postoperatively. Similarly, by categorizing individuals as having good, moderate, and poor conduit performance based on CONDUIT domain scores, we can help direct patient care and guide intervention. We determined three performance categories to interpret the scores to actionable clinical activity following common triage pathways. This decision was drawn after feedback by patients and providers. Patients with a “good” score would be encouraged to continue good behavior and track their progress, whereas those with “moderate” scores would receive targeted education and counseling about behavior and diet followed by a re-assessment, and those with a “poor” score would likely require an intervention or face-to-face evaluation (Fig. 1). Precise measurement on a continuous scale also allows for more reliable classification into these categories. Against this backdrop, the purpose of the current study was to establish performance standards or cutoff scores along the score range for the five domains and dyspnea.

## Methods

### Conduit performance descriptions

The first step of our standard setting was to prepare descriptions of performance categories, which are statements of the symptoms and health that characterize patients in discrete, clinically relevant categories. Everyone in the practice at Mayo Clinic with expertise in esophageal disorders was invited to discuss three performance categories that would be used to provide a graded level of interventions. In the previous study, we showed that the survey had five domains [5]. Based on these findings, the panel developed descriptions of “poor,” “moderate,” and “good” conduit performance for five domains separately.

### Modified and extended Angoff standard-setting method

We applied the modified and extended Angoff standard-setting method [12, 13] in the current study, which can be utilized when there are no patient data collected. The original Angoff method involves indicating whether a minimally competent person would answer an item correctly for educational tests [14]. The variation, which has been modified in attempts to improve the original method, is one of the most commonly used methods for setting cut-scores today, and has been termed modified Angoff method [12]. The current study utilized the “modified Angoff method [12],” in which panelists reviewed yes-no items and provided, for each item, an estimate of the probability of minimally good or minimally moderate patients endorsing the item. Panelists were told to consider borderline patients as those just good enough to be in the “good” and “moderate” performance category, respectively. This procedure was expanded to polytomously scored items, termed “extended Angoff method [13],” in which panelists gave an estimate of the score that borderline patients would obtain on an item with an ordinal response scale. The cut score is then computed as the sum of average ratings for individual questions. Berk [15] reported the original or the modified Angoff method as having a marked advantage in identifying the true standard because the method could be easily adapted to include consideration of error of measurement in the region of the cut score. Based on Ricker’s [12] recommendation, we included a region of indecision where a gap of one standard error of measurement in the score scale separates the performance standards.

### Panel rating and discussion

The standard setting took place in three separate meetings with each session taking between one and two hours. Multiple sessions were needed due to scheduling challenges. Discussions were facilitated by two individuals with expertise in psychometrics, quality of life measurement and survey methodology but limited clinical knowledge. These facilitators were not affiliated with any clinical department, and thus, neutral to the opinions and ratings provided by the clinicians. Panelists rated 14 items on dysphagia, seven on reflux, 10 on dumping-hypoglycemia, seven on dumping-GI, and two on pain. There were two rounds in which panelists set standards, and between the rounds there was a panel discussion. After each round, panelists’ ratings were collected and entered into a spreadsheet, and a summary of the ratings was prepared in order to provide feedback or initiate discussion on their ratings. Panelists were allowed to identify items that were content-irrelevant, or should be given higher or lower weights. They were also allowed to suggest weights to use in combining scores across items. Panelist evaluation of the process and confidence with the rating tasks was collected.

### Scoring and cut scores

After applying the weights, the estimates on polytomous items and the probability ratings on dichotomous items were summed over items in a given domain, and these sums were averaged across panelists to determine the panel cut scores. The maximum possible scores on the original summed score scales were different, depending on domains, because each domain had different numbers of dichotomous and polytomous items. Within each domain, polytomous items often had different numbers of response options, and some items’ scores were weighted higher based on recommendations by panelists. Therefore, all domains were scored on a 0–100 scale for ease of interpretation, using the following transformation: [original scale score/original scale score range]*100. Note that the minimum score on all our scales was zero. To derive the cut score, we first excluded the two most extreme ratings for each item (i.e., minimum and maximum ratings on an item). For each item, the ratings were averaged across remaining panelists. The final cut score was the sum of these averages. The standard error of the cuts was computed using the Central Limit Theorem, with the equation,

$$ {S}_E=\frac{S}{\sqrt{n}} $$

where *S* is the standard deviation of the cut scores across *n* number of judges. Note that we removed two most extreme ratings (minimum and maximum), so the *S*_*E*_ was computed with *n-*2 ratings [12]. A lower standard error is desirable because it denotes better agreement among the judges and less uncertainty about where the true cut scores should lie.

We compared the standard errors of the cut scores without applying weights and with applying weights. The cut scores with smaller standard errors were chosen.

## Results

### Conduit performance descriptions

A panel composed of 12 clinicians with expertise in esophageal disorders met to discuss three performance categories. According to panelists, patients have good conduit performance when they communicate hardly any problems on the CONDUIT. Patients are judged to have moderate conduit performance when they communicate some problems. These patients experience mild to moderate symptoms after esophagectomy, which may improve after behavior changes. Patients are judged to have poor conduit performance when their conduits do not function regardless of patients’ symptom-management behavior. Panelists suggested that how we evaluate surgery-related pain should change as time passes. No pain to mild pain is regarded as good performance regardless of time, and severe pain is regarded as poor performance at any time. Moderate pain is indicative of moderate conduit performance within six months since surgery. However, moderate pain is indicative of poor conduit performance six or more months post-surgery. Therefore, if a patient had a surgery more than six months ago, and her pain score falls in the “moderate” category, her report would say “severe” instead of “moderate.”

### Standard setting

#### Panel composition, training, and ratings

The panel was composed of 10 physicians, five nurse practitioners or physician assistants, and one clinical nurse specialist, representing the Divisions of Surgery (*n* = 9), Medical Oncology (*n* = 3), Gastroenterology (*n* = 3), and Nursing (*n* = 1). They cared for patients with esophageal reconstruction for 16.3 years on average (SD = 11.1 years). After reviewing the descriptions of conduit performance categories and definition of borderline patients, the panel was trained on modified and extended Angoff methods with three dichotomous and three polytomous items drawn from the actual survey. At this initial meeting, they provided ratings for borderline good and moderate patients on 14 dysphagia items, seven reflux items, 10 hypoglycemia dumping items, seven gastrointestinal dumping items, and two pain items, producing 78 ratings per panelist under two facilitators’ guidance. The subsequent panel rated four additional items on reflux symptoms, two additional on dumping-hypoglycemia, two reworded items on dumping-GI, and the MRC breathlessness scale. This latter panel had participated in the earlier meeting, and was composed of six physicians and four nurse practitioners or physician assistants.

#### Round 1 evaluation

The panel discussed their ratings on each item after the Round 1 rating task, and completed Round 1 evaluation. Panels generally agreed that they understood the purpose of the study (94%), the training on the standard-setting method gave them the information they needed to complete their assignments (81%), and that they understood the concept of the borderline patient (94%). However, some (19%) mentioned the difficulty of coming up with percentages of borderline patients (especially borderline moderate patients) endorsing the item. All panelists rated that experience with patients was influential on item ratings. Perception of the severity of symptoms that the items were measuring (94%), and the description of borderline patients and the performance descriptions (81%) were influential in their ratings.

#### Final evaluation

After the opportunity to change their ratings in the Round 2 session, panelists completed their final evaluation (Table 1). We assumed an interval scale for Strongly Disagree (1), Disagree (2), Agree (3), and Strongly Agree (4), and computed the mean of the ratings. In general, they agreed that it was beneficial to have an opportunity for discussion and to review feedback (mean = 3.2); that the opportunity to provide a second round of ratings helped them feel more confident about their final ratings (mean = 3.0); that they felt engaged in the process (mean = 3.2); and that they felt comfortable sharing their ideas with the other panelists during the discussions (mean = 3.4). They gave slightly lower ratings than “Agree” on statements that this standard-setting process will produce fair cut scores (mean = 2.9), and they were comfortable defending this process to their peers (mean = 2.9). Panelists who checked “Disagree” on the statements commented that it was hard to judge how the cut scores would be useful at this stage, and that it was easier to rate polytomous items compared to dichotomous items; difficulty with dichotomous items was attributed to identifying the percentages of borderline patients. On the scale from “Not Useful,” “Useful,” and “Very Useful,” panelists (91%) rated that referencing the performance descriptions and the large group discussion after the Round 1 task were “Useful” or “Very Useful.”

**Table 1:** Responses to Final Evaluation

	% Strongly Agree	% Agree	% Disagree/ Strongly Disagree
1. I understood the purpose of the study.	27%	73%	0%
2. The instructions and explanations provided by the facilitator were clear.	9%	73%	18%
3. The training on the standard-setting method gave me the information I needed to complete my assignment.	9%	91%	0%
4. The Performance Descriptions that were developed prior to the meeting were accurate.	18%	55%	27%
5. I understood the concept of the borderline patient.	36%	64%	0%
6. The Performance Descriptions helped me determine how to rate each item.	18%	73%	9%
7. It was beneficial to have an opportunity for discussion and to review feedback.	36%	46%	18%
8. The opportunity to provide a second round of ratings (i.e., round 2) helped me feel more confident about my final ratings.	18%	64%	18%
9. I felt engaged in the process.	18%	82%	0%
10. I felt comfortable sharing my ideas with the other panelists during the discussions.	45%	55%	0%
11. I am confident this standard-setting process will produce fair cut scores.	9%	73%	18%
12. I would be comfortable defending this process to my peers.	18%	55%	27%
	Very influential	Influential	Not influential
13. My perception of the severity of symptoms that the items were measuring	27%	73%	0%
14. The Performance Descriptions	18%	64%	18%
15. The average ratings of other panelists	9%	73%	18%
16. Large group discussion after Round 1	9%	73%	18%
17. My experience with patients	55%	45%	0%
	Very useful	Useful	Not useful
18. Practicing the procedure with real items prior to beginning the actual rating task	18%	37%	45%
19. Referencing the Performance Descriptions	9%	82%	9%
20. Large Group discussion after Round 1	27%	64%	9%

### Conduit performance descriptions

After the first standard-setting meeting, panelists provided feedback that some of the performance descriptions were circular (e.g., if a patient has a good conduit emptying score, then his/her conduit score is “good”), or symptoms were not separated from behavior (e.g., if a patient has to elevate head of bed, then the patient is “good” in reflux). Panelists also commented on weight maintenance. Therefore, we improved the conduit performance descriptions by making each statement clearer and adding two additional domains for reporting and tracking purpose: dyspnea and weight maintenance. Table 2 presents the consensus conduit performance descriptions developed by panelists.

**Table 2:** Conduit Performance Descriptions

	Good	Moderate	Poor (frequent or severe)
Dysphagia	-Able to eat with no limitations -Able to swallow most soft food -Able to swallow all liquids -Some difficulty with certain hard solid food (e.g., bread and meat)	-Difficulty with swallowing soft food -Able to swallow all liquids	-Difficulty swallowing liquids or saliva
Reflux (heartburn and/or acid regurgitation)	-Patient has no reflux symptoms -No reflux symptoms when sleeping with the head elevated -No reflux symptoms with medication	-Patient has mild symptoms when sleeping with head elevated -Patient has mild reflux symptoms with medication	-Patient has aspiration -Patient has constant reflux symptoms -Patient has moderate/severe reflux symptoms with medication -Patient has moderate/severe reflux symptoms when sleeping with head elevated
Dumping-Hypoglycemia within three hours of eating	-Patient experiences none or a few symptoms^a^	-Patient experiences several symptoms^a^ -Some improvement with behavior changes^b^	-Patient experiences many symptoms^a^ -No improvement with behavior changes^b^
Dumping-GI within three hours of eating	-Patient experiences none or a few symptoms^c^	-Patient experiences several symptoms^c^ -Some improvement with behavior changes^b^	-Patient experiences many symptoms^c^ -No improvement with behavior changes^b^
Surgery Related Pain	-Pain is mild	-Within six months of surgery pain is moderate	- Within six months of surgery pain is severe -After six months pain is moderate to severe
Dyspnea	-Breathlessness is mild	-Breathlessness is moderate	-Breathlessness is severe
Maintaining Weight	-weight has stabilized -weight is changing as expected given the patient’s phase of recovery - less than 10% difference from post-conduit surgery target weight	- minor weight change continues after time when patient should have stabilized - within 10%–20% of post- conduit surgery target weight	- significant weight change continues after time when patient should have stabilized - more than 20% difference from post- conduit surgery target weight

^a^Dumping-hypoglycemia symptoms include shock, fainting/loss of consciousness/ passing out, dizziness, breathlessness/shortness of breath, weakness, exhaustion/desire to lie down due to weakness, sleepiness/drowsiness, palpitations, restlessness, and headache

^b^Behavior changes include avoiding sugar or carbohydrates, taking medication, or eating 5–6 meals a day instead of 3 meals a day

^c^Dumping-GI symptoms include nausea or feeling like wanting to throw up, abdominal fullness/abnormal collection of gas in the abdomen, rumbling sound from your stomach or intestines, belching/burping, and diarrhea

### Follow-up meeting

The second standard setting took place in two separate meetings with five panelists in each, which lasted about 45 min. All 10 panelists were familiar with the method because they participated in the first meeting. Among the nine items they rated, there was a single-item MRC breathlessness scale, two items in dumping-GI that had been reworded, and six new items (four in reflux, two in dumping-hypoglycemia) that were written after the first standard-setting meeting. They were allowed to change their ratings after the first round. The follow-up meeting incorporated real-time averaging, recording, and sharing of results as the discussion proceeded. Panelist evaluations were not collected from this meeting.

### Final number of items and weighting

After this iterative process, the final numbers of items were 13 for dysphagia, 11 reflux, 12 dumping-hypoglycemia, 7 dumping-GI, and 2 pain. We had 13 items for dysphagia, because we dropped an item on time taken to eat a meal following the panelists’ opinion that eating time is not only influenced by conduit performance but one’s lifestyle. The panel additionally recommended that the following items be weighted twice as high in their respective domains, given the importance of these items for categorizing patients into clinically relevant groups: severity of trouble swallowing (dysphagia), whether heartburn awakens a patient at night, aspiration, whether acid regurgitation causes the patient to cough or his/her voice to become hoarse (reflux), fainting and loss of consciousness due to dumping (dumping-hypoglycemia), and whether one experienced dumping syndrome symptoms with each meal (dumping-GI and dumping-hypoglycemia). One question on trouble swallowing liquids was weighted three times higher (dysphagia). No pain items were weighted.

### Cut scores

For the three domains that had new item ratings in the second standard-setting meeting, we derived the new cuts by summing the cut scores from the earlier and newer items and mapping them to a 0–100 reporting scale. The new standard error of the cut scores for these domains was computed using the Satterthwaite approximation of the standard errors,

$$ {S}_E=\sqrt{\frac{s_1^2}{n_1}+\frac{s_2^2}{n_2}} $$

where ${s}_1^2$ is the variance of the ratings from the initial meeting excluding the minimum and maximum ratings from the meeting, *n*_1_ is the number of judges from the initial meeting minus two, ${s}_2^2$ is the variance of the ratings from the additional meeting excluding the minimum and maximum ratings from this meeting, and *n*_2_ is the number of judges from this additional meeting minus two.

The cut scores and their standard errors on a 0–100 scale are presented in Table 3. We also compared the cut scores with and without weights recommended by panelists. With or without weighting, the good/moderate cuts were found between scores of 7.2 and 20.8, and moderate/poor cuts between 37.9 and 64.3. Standard errors of the cut scores became larger when weights were applied for dysphagia and dumping-GI (moderate/poor cut), whereas they became smaller for reflux and dumping-hypoglycemia. Therefore, we maintained the cut scores with weights for only reflux and dumping-hypoglycemia.

**Table 3:** Comparison of Cut Scores With and Without Weighting Some Items

	good/moderate cut	moderate/poor cut	SE of good/mod cut	SE of mod/poor cut
Dysphagia				
no weighting	20.1	62.9	1.8	1.8
weighting	19.3	57.6	2.4	3.2
Reflux				
no weighting	19.0	54.2	3.2	5.5
weighting	17.0	50.1	3.0	5.5
Dumping-Hypoglycemia				
no weighting	7.6	40.8	2.0	4.6
weighting	7.2	37.9	1.8	4.4
Dumping-GI				
no weighting	12.0	42.8	4.1	3.0
weighting	11.2	40.6	4.0	3.8
Pain				
no weighting	20.8	64.3	2.1	2.0
weighting	NA	NA	NA	NA
Dyspnea				
no weighting	1.4	3.3	0.2	0.1
weighting	NA	NA	NA	NA

*SE*-standard error

*NA*-not applicable

Lastly, for the domain dyspnea, a previously established measure, MRC breathlessness scale, was used, which reports scores on integer scale from 1 to 5. Their scores, as well as cut scores, were not transformed to 0–100. Table 3 presents cut scores on MRC breathlessness scale. The results suggested that the score of one is considered good, the scores of two and three are moderate, and the scores of four and five are poor.

## Discussion

The current study utilized Angoff methods to set good/moderate and moderate/poor cut scores for each of the five novel domains of the CONDUIT questionnaire and the MRC breathlessness scale. We described how the panel developed performance categories, were trained, and provided ratings, which were then aggregated to come up with cut scores. We also described in detail how panelists suggested weights to certain items, or decided to add or drop items. The items that were dropped or reworded later were in pilot stage, so we did not describe them in detail. We present the sample items with abbreviated stems and response options from the current CONDUIT Report Card in the Additional file 1. Good/moderate cuts were 20.1 for dysphagia, 17.0 for reflux, 7.2 for dumping-hypoglycemia, 12.0 for dumping-GI, and 20.8 for pain. Moderate/poor cuts were 62.9 for dysphagia, 50.1 for reflux, 37.9 for dumping-hypoglycemia, 42.8 for dumping-GI, and 64.3 for pain. The good/moderate cut for dyspnea on MRC breathlessness scale was 1.4, and the moderate/poor cut 3.3.

This is the first standard-setting study for the scores on the CONDUIT. Validated cut scores are useful for clinicians to efficiently place patients in clinically relevant groups to facilitate tailored symptom management. The methods used in this study are partially sensitive to patient performance. Because Angoff cut scores are set independent of patient data, they may not be realistic. Therefore, utilization of impact data such as percentage of patients who end up in each category can help discern whether we set realistic standards. In addition, the performance standards and cut scores should be evaluated in relation to performance in real-life settings. This can yield estimates of correctly and incorrectly classifying patients when performance information is available.

There is no particular criticism or concern with the Angoff method being statistically sound. One limitation of our study is in the way we combined the standard errors from two standard-setting meetings for domains with new or reworded items. We assumed independence of the observations among expert ratings, although all experts who participated in the second meeting were also present in the first meeting. This was because the ratings were de-identified from each meeting. Because some raters can have tendencies to give higher or lower ratings, the standard errors of our cut scores may have been overestimated.

## Conclusion

Standard setting is, in large part, a judgmental process. Therefore, in this study, we described, in detail, selection and training of panelists, and the processes they were asked to follow in order to support defensibility of cut scores. Initial studies established a set of domains. This study is the second and essential step in the process of validation of a tool that can be used to manage patients after esophagectomy and compare patient-reported outcomes. Further prospective validation studies are underway to compare cut scores from this study and clinicians’ judgments and further refine the categorization. Once complete, this tool can be utilized by clinicians and patients to improve techniques, target education or intervention, and compare outcomes.

## Additional file

Additional file 1: Sample items with abbreviated stems and abbreviated response options in the five domains from the CONDUIT Report Card. (DOCX 19 kb)

## Abbreviations

CONDUIT: Mayo Clinic Esophageal Conduit Outcomes Noting Dysphagia/Dumping, and Unknown Outcomes with Intermittent Symptoms Over Time After Esophageal Reconstruction (CONDUIT) Report Card
GI: Gastrointestinal
MRC: Medical Research Council
QOL: Quality of Life

## References

[1] Chang YL, Tsai YF, Chao YK, Wu MY (2016). Quality-of-life measures as predictors of post-esophagectomy survival of patients with esophageal cancer. Qual Life Res Int J Qual Life Asp Treat Care Rehab.

[2] Chang YL, Tsai YF, Wu YC, Hsieh MJ (2014). Factors relating to quality of life after esophagectomy for cancer patients in Taiwan. Cancer Nurs.

[3] Viklund P, Wengstrom Y, Rouvelas I, Lindblad M, Lagergren J (2006). Quality of life and persisting symptoms after oesophageal cancer surgery. Eur J Cancer.

[4] Basch E, Deal AM, Kris MG, Scher HI, Hudis CA, Sabbatini P, Rogak L, Bennett AV, Dueck AC, Atkinson TM (2016). Symptom monitoring with patient-reported outcomes during routine Cancer treatment: A randomized controlled trial. J Clin Oncol Off J Am Soc Clin Oncol.

[5] Lee, M. K., Yost, K. J., Pierson, K. E., & Blackmon, S. H. Patient-reported outcome domains for the esophageal CONDUIT Report Card: A prospective trial to establish domains. Health and Quality of Life Outcomes. 10.1186/s12955-018-1023-7. 10.1186/s12955-018-1023-7 PMC6180437 30305083

[6] Stenton C (2008). The MRC breathlessness scale. Occup Med.

[7] Eckardt VF, Aignherr C, Bernhard G (1992). Predictors of outcome in patients with achalasia treated by pneumatic dilation. Gastroenterology.

[8] Vantrappen G, Hellemans J (1980). Treatment of achalasia and related motor disorders. Gastroenterology.

[9] Javle M, Ailawadhi S, Yang GY, Nwogu CE, Schiff MD, Nava HR (2006). Palliation of malignant dysphagia in esophageal cancer: A literature-based review. J Support Oncol.

[10] Jeansonne LO, White BC, Pilger KE, Shane MD, Zagorski S, Davis SS, Hunter JG, Lin E, Smith CD (2007). Ten-year follow-up of laparoscopic Heller myotomy for achalasia shows durability. Surg Endosc.

[11] Arain MA, Peters JH, Tamhankar AP, Portale G, Almogy G, DeMeester SR, Crookes PF, Hagen JA, Bremner CG, DeMeester TR (2004). Preoperative lower esophageal sphincter pressure affects outcome of laparoscopic esophageal myotomy for achalasia. J Gastrointest Surg.

[12] Ricker KL (2006). Setting cut-scores: A critical review of the Angoff and modified Angoff methods. Alberta J Educ Res.

[13] Hambleton RK, Plake BS (1995). Using an extended Angoff procedure to set standards on complex performance assessments. Appl Meas Educ.

[14] Angoff WH, Thorndike RL (1971). Scales, norms and equivalent scores. Educational Measurement.

[15] Berk RA (1986). A consumer’s guide to setting performance standards on criterion-referenced tests. Rev Educ Res.

