# Supplementary material for: Standard setting for a novel esophageal conduit questionnaire: CONDUIT Report Card
Source: J Patient Rep Outcomes. 2018 Oct 24;2:51. doi: 10.1186/s41687-018-0073-2 (PMC6200834; doi:10.1186/s41687-018-0073-2)
Supplement: Supplementary file 1 — Sample items with abbreviated stems and abbreviated response options in the five domains from the CONDUIT Report Card. (DOCX 19 kb) [file 41687_2018_73_MOESM1_ESM.docx]

**Additional file 1** Sample items with abbreviated stems and abbreviated response options in the five domains from the CONDUIT Report Card

**Dysphagia**

1. Frequency of difficulty swallowing

Never to Daily

2. Severity of trouble swallowing ^1^

Doesn’t bother me at all to Very severe-greatly affects my lifestyle

3. Swallowing in general

Able to eat anything to Unable to swallow anything

4. How long solid food got stuck

Didn’t get stuck to 5 minutes or more

5. Problems swallowing liquids ^2^

No and Yes

6. Problems swallowing solid foods

No and Yes

**Reflux**

1. Frequency of heartburn

Never to Daily

2. Waking at night due to heartburn ^3^

No and Yes

3. Voice becoming hoarse due to acid regurgitation? ^4^

No and Yes

4. Coughing due to acid regurgitation ^5^

No and Yes

5. Aspirated

Never to Daily

**Dumping-hypoglycemia**

Symptoms of “dumping syndrome” within 3 hours of eating or receiving tube feeding

1. Shock

No and Yes

2. Fainting ^6^

No and Yes

3. Dizziness

No and Yes

4. Frequency of above symptoms

Never to Daily

5. Symptoms with each meal ^7^

No and Yes

**Dumping-Gastrointestinal symptoms**

Symptoms of “dumping syndrome” within 3 hours of eating or receiving tube feeding

1. Nausea

No and Yes

2. Abdominal fullness

No and Yes

3. Diarrhea

No and Yes

4. Frequency of above symptoms

Never to Daily

7. Symptoms with each meal ^8^

No and Yes

**Pain**

1. Pain on average

No pain to Worst imaginable pain

2. Frequency of pain

No pain from surgery to All of the time

^1^ Panel suggested weighting this item twice as high in dysphagia, but did not get weighted because of larger standard errors in cut scores

^2^ Panel suggested weighting this item three times as high in dysphagia, but did not get weighted because of larger standard errors in cut scores.

^3, 4, 5^ Panel suggested weighting these items twice as high in reflux.

^6, 7^ Panel suggested weighting these items twice as high in dumping-hypoglycemia.

^8^ Panel suggested weighting this item twice as high in dumping-gastrointestinal symptoms, but did not get weighted because of larger standard errors in cut scores.

©2018 Mayo Foundation for Medical Education and Research; all rights reserved.
